# The algorithmic patient: on the ontological disruption of selfhood, suffering, and healing in the age of artificial intelligence

**DOI:** 10.3389/fpsyg.2026.1809692

**Published:** 2026-05-01

**Authors:** Changiz Mohiyeddini

**Affiliations:** Department of Foundational Medical Studies, Oakland University William Beaumont School of Medicine, Rochester, MI, United States

**Keywords:** algorithmic epistemology, artificial intelligence, clinical phenomenology, digital health, embodied cognition, health psychology, moral psychology, ontology of illness

A Critical Perspective for the Pioneers & Pathfinders Research Topic, Frontiers in Psychology−15th Anniversary Special Edition.

## 1 Introduction: the uninvited guest at the bedside

“*The question is not whether machines can think, but whether humans can continue to think about themselves in the same way once machines appear to.”*

—Adapted from Weizenbaum, 1976

As Frontiers in Psychology marks its 15th anniversary, the discipline of health psychology stands at what may be its most consequential inflection point. The past decade has seen artificial intelligence transition from a distant computational curiosity to an omnipresent force reshaping how illness is detected, how health behaviors are monitored, and how clinical decisions are made. Large language models offer therapeutic conversation. Wearable algorithms predict depressive episodes before patients report symptoms. Machine learning classifies disease trajectories with an accuracy that humbles clinical intuition. The question that every health psychologist must now confront is not whether AI will transform the field—it already has—but whether the field possesses the conceptual vocabulary to understand what is being transformed.

This article contends that it does not. Not yet.

The prevailing discourse around AI in health psychology has been dominated by two symmetrical errors: uncritical techno-optimism, which treats AI as an efficiency multiplier for existing paradigms, and reflexive techno-skepticism, which dismisses AI as a threat to humanistic care. Both positions share a common flaw—they treat AI as a *tool*, an instrument external to the phenomena of health, illness, and healing that health psychology studies. I argue, by contrast, that AI is better understood as an *ontological disruption*: a force that alters the very categories through which we apprehend what it means to be ill, to suffer, to seek help, and to recover.

The philosophical stakes are immense. If an algorithm can predict that a person will develop major depressive disorder 18 months before any subjective experience of sadness, what does this mean for the phenomenological primacy of lived experience that grounds our discipline? If a chatbot can produce therapeutic outcomes comparable to human therapists in randomized controlled trials, does this vindicate or demolish the centrality of the therapeutic relationship? If digital nudging systems can modify health behaviors more effectively than any motivational intervention, what remains of the concept of autonomous self-determination that health psychology has championed since its inception?

These are not hypothetical provocations. They are empirical realities pressing against a theoretical infrastructure that was designed for a different world. The biopsychosocial model (Engel, 1977), self-determination theory (Deci and Ryan, 1985), the health belief model (Rosenstock, 1966), the theory of planned behavior (Ajzen, 1991)—these frameworks were conceived in an era when the primary agents of health and illness were understood to be biological organisms embedded in social contexts, making decisions through psychological processes accessible (at least in principle) to introspection and interpersonal dialogue. None of these frameworks anticipated a world in which a non-conscious computational system could become a constitutive element of the illness experience itself.

What follows is an attempt to think through—not around—this disruption. I offer neither a literature review nor an empirical report, but a sustained philosophical argument organized around four disruptions that AI introduces into the conceptual architecture of health psychology. My purpose is not to resolve these tensions but to make them visible, and to propose that health psychology's future depends on its willingness to become, in part, a philosophical discipline—one capable of asking questions about the nature of persons, the meaning of suffering, and the ethics of care that exceed the methodological conventions of our field.

## 2 The first disruption: the phenomenology of illness and the problem of algorithmic foreknowledge

Health psychology has always maintained, implicitly or explicitly, that illness is not merely a biological event but an *experiential phenomenon*. The discipline inherited from Canguilhem (1966) the insight that disease is not a deviation from a statistical norm but a disruption of the individual's capacity to engage with their world. From Merleau-Ponty (1945), it absorbed the understanding that the body is not an object but a subject—that illness transforms the body from a transparent medium of action into an obstruction, a source of pain, an alienated other. The phenomenological tradition insists that illness is constituted in the first person: it is something *undergone*, not something merely detected.

AI destabilizes this foundational commitment by introducing what I shall call *algorithmic foreknowledge*—the capacity to identify pathological processes before they cross the threshold of subjective awareness. Consider the empirical landscape. Machine learning algorithms now predict the onset of psychotic episodes from smartphone keystroke dynamics (Zulueta et al., 2018), detect early-stage Parkinson's disease from voice recordings (Tracy et al., 2020), and identify cardiovascular risk from retinal scans with accuracy rivaling or exceeding traditional biomarkers (Poplin et al., 2018). In each case, the algorithm “knows” something about the person's body that the person does not know—and may never come to know in the same way.

This is not merely a matter of earlier detection. It is a restructuring of the temporal architecture of illness. In the traditional phenomenological account, illness unfolds through a sequence: disruption of bodily transparency, attentional shift toward the body, interpretation of symptoms, narration of illness, and engagement with healthcare systems. Each stage involves the active participation of the ill person as a meaning-making subject. Algorithmic foreknowledge collapses this sequence. The person is informed—by an app, a wearable device, a clinical report generated by neural networks—that they are ill (or will become ill) before they have undergone any disruption of their lived experience. They are patients before they are sufferers.

The philosophical implications are vertiginous. If illness can be constituted algorithmically prior to experiential disruption, then the phenomenological primacy of lived experience—the bedrock upon which much of health psychology's person-centered ethos is built—is no longer self-evident. A new category of personhood emerges: the *algorithmically ill*, individuals who are pathologized by computation in the absence of suffering. How does health psychology address someone who has been told by a machine that they will develop depression but who currently feels well? Is this person a patient? A pre-patient? A data point? Our existing models have no adequate answer.

This is not a merely academic concern. The psychological consequences of algorithmic foreknowledge are already materializing in clinical practice. Research on incidental findings in genomic screening has demonstrated that probabilistic health information can generate sustained anxiety, hypervigilance, and identity disruption even in the absence of disease (Broadstock et al., 2000; Oliveri et al., 2018). When the source of such information shifts from genetic counselors—who communicate within an intersubjective relationship—to algorithmic systems that deliver verdicts through smartphone notifications, the potential for ontological disorientation intensifies. The person's body becomes, in Heidegger's (1927) sense, *unheimlich*—uncanny, no longer at home—not because of felt illness, but because of computational inference about unfelt pathology.

Health psychology urgently needs a phenomenology of algorithmic illness—a systematic account of what it means to experience oneself as ill-according-to-the-machine. Such an account would need to grapple with the paradox that the person's authority over their own experience—their epistemic privilege as the one who undergoes their body—is being quietly overridden by systems that claim to know the body better than the body knows itself.

## 3 The second disruption: the therapeutic relationship and the Turing trap

No concept is more central to health psychology's clinical identity than the therapeutic relationship. From Rogers' (1957) articulation of the necessary and sufficient conditions for therapeutic change to the vast contemporary literature on therapeutic alliance (Flückiger et al., 2018), the field has consistently maintained that healing occurs within an interpersonal encounter characterized by empathy, unconditional positive regard, and genuine presence. The therapeutic relationship is not merely the vehicle for delivering interventions; it is, on many accounts, the intervention itself.

AI-driven therapeutic systems—large language model chatbots, virtual agents, app-based cognitive behavioral therapy programs—now challenge this assumption with uncomfortable empirical force. A growing body of evidence suggests that automated systems can produce clinically meaningful improvements in depression, anxiety, and health behavior change (Fitzpatrick et al., 2017; Fulmer et al., 2018). Some users report feeling more comfortable disclosing sensitive health information to non-human agents precisely because there is no fear of interpersonal judgment (Lucas et al., 2014). The machine's inability to judge is experienced, paradoxically, as a form of safety.

The discipline is thus caught in what I call the *Turing Trap*: the temptation to evaluate AI's therapeutic legitimacy by whether it can mimic the outputs of human therapy—symptom reduction, behavioral activation, reported satisfaction—rather than asking whether the process involved constitutes therapy in any meaningful sense. If we define therapy solely by its measurable outcomes, then a well-designed chatbot is a therapist. If we define therapy as an intersubjective encounter between conscious beings, then no computational system, however sophisticated, can be a therapist—because there is, in Nagel's (1974) formulation, nothing it is like to be the machine. There is no experience on the other side of the therapeutic encounter. The empathy is simulated. The regard is programmatic. The presence is absence.

This distinction matters enormously for health psychology, and not only for philosophical reasons. The therapeutic relationship in health contexts serves functions that transcend symptom management. It provides *witness* to suffering—the existential validation that one's pain has been received by another consciousness. It offers a model of *relational coherence* in which the disruptions of illness are metabolized through the steady presence of another. It instantiates a form of *moral recognition*—the acknowledgment that the suffering person is a subject, not merely a collection of symptoms to be optimized. None of these functions can be performed by a system that does not possess consciousness, subjectivity, or moral agency, regardless of how convincingly it simulates them.

And yet we must be honest about what the evidence reveals. If patients improve—if their distress diminishes, their health behaviors change, their quality of life increases—can we dismiss this as merely the placebo effect of a well-designed interface? Doing so would betray the very empiricism that health psychology claims to honor. The uncomfortable truth is that the evidence may be revealing something about the nature of therapeutic change that challenges our cherished assumptions: that much of what we attributed to the uniquely human qualities of the therapist may, in fact, be attributable to the structure of the intervention, the regularity of contact, the provision of psychoeducation, and the scaffolding of self-monitoring—all of which can be delivered computationally.

Health psychology must hold this tension without collapsing into either pole. We need what the philosopher Ricoeur (1970) called a *hermeneutics of suspicion* directed at our own assumptions about what makes therapy work, combined with a *hermeneutics of faith* that refuses to reduce the encounter between suffering persons to an information-processing problem. The question is not whether AI can produce therapeutic outcomes—it manifestly can—but what is *lost* in the transaction, and whether what is lost matters.

## 4 The third disruption: autonomy, self-determination, and the architecture of digital nudging

Self-determination theory (SDT; Ryan and Deci, 2000) has provided health psychology with one of its most powerful and empirically supported frameworks. The theory's insistence that autonomous motivation—behavior experienced as volitional, self-endorsed, and congruent with one's values—is the most sustainable driver of health behavior change has shaped decades of intervention design. The distinction between autonomous and controlled motivation has become axiomatic: we seek to help people *want* to be healthy, not merely to *comply* with health directives.

AI-driven health technologies introduce a radical ambiguity into this framework. Consider the architecture of a contemporary digital health platform. Machine learning algorithms analyze behavioral data—step counts, sleep patterns, dietary inputs, mood logs—to generate personalized recommendations timed to moments of maximum psychological receptivity. Reinforcement learning systems calibrate the frequency, framing, and emotional valence of health messages to maximize adherence. The entire system is designed, at the level of its computational architecture, to shape behavior—and it does so by operating on the very psychological processes that SDT identifies as the locus of autonomous functioning.

The philosophical problem is this: when a person changes their health behavior in response to an algorithmically optimized intervention that has been precisely calibrated to exploit their psychological vulnerabilities and strengths—delivered at the exact moment when their defenses are lowest and their receptivity is highest—is the resulting behavior autonomous? The person may *experience* it as autonomous. They may report, on validated self-report measures, that their behavior feels self-determined. But the phenomenology of autonomy may be dissociable from its reality. The most effective form of control, as Marcuse (1964) observed, is the one that is experienced as freedom.

This is the problem of what I term *algorithmic heteronomy masked as autonomy*. Health psychology's commitment to self-determination was forged in an era when the primary threats to autonomous motivation were identifiable and external: coercive medical advice, controlling social environments, internalized cultural imperatives. The algorithmic threat is qualitatively different because it is *adaptive*—it learns the person's motivational architecture and adjusts its influence in real time. It does not override autonomy through force; it *metabolizes* autonomy, incorporating the person's own values and preferences into its persuasive strategy. The person is not coerced. They are *curated*.

SDT's architects have begun to address these concerns (Ryan and Deci, 2020), and the broader literature on digital wellbeing has raised important questions about techno-autonomy (Burr et al., 2020). But health psychology as a discipline has not yet confronted the full depth of the challenge. We need a theory of autonomy that can distinguish between genuine self-determination and algorithmically manufactured consent—and we need empirical methods capable of detecting the difference. This is not merely a theoretical desideratum; it has immediate practical implications for the ethical design of digital health interventions, the validity of informed consent procedures in AI-mediated care, and the interpretation of health behavior change outcomes in algorithmically saturated environments.

## 5 The fourth disruption: the moral structure of care and the question of algorithmic responsibility

Health psychology is, at its core, a moral enterprise. Not in the prescriptive sense of imposing values, but in the deeper sense that its fundamental orientation—toward the alleviation of suffering, the promotion of wellbeing, the empowerment of persons to live in accordance with their own conception of the good—presupposes a moral anthropology. It presupposes that human beings are ends in themselves, that suffering matters, that the relationship between caregiver and patient carries moral weight, and that health is not merely a biological state but a dimension of human flourishing.

AI threatens to hollow out this moral structure from within. Not because AI systems are immoral, but because they are *amoral*—they optimize objective functions without any comprehension of the moral significance of what they are doing. When an algorithm triages patients for psychological intervention, it is maximizing a mathematical function, not exercising moral judgment. When it identifies a patient as high-risk for suicide, it is not *concerned*—it is classifying. The appearance of care is generated by the system; the reality of care is absent.

The problem extends to questions of responsibility. In traditional health psychology practice, the chain of moral responsibility is (relatively) clear. A clinician assesses a patient; the clinician makes a judgment; if the judgment is wrong, the clinician bears moral and professional responsibility. AI disrupts this chain by distributing agency across a network of actors—the developers who designed the algorithm, the data scientists who trained it, the institution that deployed it, the clinician who followed its recommendation, and the patient who consented to its use—none of whom individually bears the kind of concentrated moral responsibility that the traditional model assumes.

This diffusion of responsibility has profound implications for the patient's experience of being cared for. Levinas (1961) argued that the ethical relation begins with the *face of the other*—the irreducible encounter with another consciousness that makes a demand upon me. The patient who presents to a health psychologist is making such a demand: *See me. Hear my suffering. Take responsibility for helping me*. An algorithm cannot receive this demand because it has no face. It has an interface. And the interface, however empathically designed, is not a locus of moral subjectivity. It cannot be held accountable. It cannot be moved by suffering. It cannot, in any meaningful sense, *care*.

Health psychology must grapple with the possibility that the algorithmic transformation of care is not merely a change in delivery mechanism but a change in the *ontological character* of what is delivered. A world in which health care is increasingly mediated by amoral computational systems is a world in which the moral texture of the care relationship is fundamentally altered—even if the clinical outcomes remain statistically equivalent. The question that health psychology must learn to ask is not only “Does it work?” but “What kind of world are we creating, and what kind of persons are we becoming, when we submit our suffering to algorithmic adjudication?”

## 6 Toward an ontologically informed health psychology

The four disruptions I have outlined—to the phenomenology of illness, the therapeutic relationship, the autonomy of health behavior, and the moral structure of care—are not independent challenges. They are manifestations of a single underlying transformation: the entry of a new kind of agent into the ecology of human health. An agent that reasons without understanding, predicts without experiencing, and intervenes without caring. The challenge for health psychology is not to accept or reject this agent, but to develop a conceptual framework adequate to its presence.

I propose that what is needed is an *ontologically informed health psychology*—a version of the discipline that takes seriously the question of what kind of being the patient is, what kind of being the healer is, and what kind of relationship is possible between them in a world increasingly populated by entities that defy the traditional categories of person and tool. Such a discipline would be characterized by several commitments.

### 6.1 Phenomenological vigilance

An ontologically informed health psychology would insist on the continued primacy of the first-person perspective in understanding illness, while honestly acknowledging that this perspective is no longer the sole epistemic authority on the body. It would develop methods for studying how algorithmic foreknowledge transforms the experience of embodiment, and it would resist the reduction of health to biomarker optimization. It would ask not only what the data say about the patient, but what it is like to be the patient about whom the data speak.

### 6.2 Relational realism

Such a discipline would maintain, against the evidence of equivalent outcomes, that there is something irreducible about the intersubjective encounter between conscious beings that cannot be captured by computational simulation—while remaining empirically rigorous about specifying what that something is. It would invest in research that distinguishes the active ingredients of therapeutic change from the relational context in which they are delivered, precisely so that we can articulate—rather than merely assert—what is at stake when the context becomes algorithmic.

### 6.3 Autonomy literacy

An ontologically informed health psychology would equip both practitioners and patients with the conceptual tools to distinguish autonomous health behavior from algorithmically curated behavior. It would develop what might be called *autonomy literacy*—the capacity to recognize the mechanisms by which digital systems shape motivation, and to make genuinely informed choices about whether and how to engage with AI-mediated health interventions. This is not digital literacy in the conventional sense; it is a philosophical competency, a capacity for critical self-reflection about the conditions under which one's choices are one's own.

### 6.4 Moral clarity

Finally, an ontologically informed health psychology would refuse to outsource the moral dimensions of care to technological efficiency. It would insist that the question of what we owe to suffering persons—and what suffering persons are entitled to demand of those who claim to help them—is a question that precedes and exceeds any algorithmic computation. It would develop ethical frameworks specifically tailored to the AI-mediated health context, frameworks that specify who bears responsibility when algorithmic decisions go wrong, what forms of consent are meaningful in algorithmically saturated environments, and how to preserve the moral substance of the care relationship in an era of computational mediation.

## 7 Conclusion: the questions that no algorithm can answer

“*We do not err because truth is difficult to see. It is visible at a glance. We err because the lie is more comfortable.”*

—Alexander Solzhenitsyn

Fifteen years ago, when Frontiers in Psychology published its inaugural issue, the notion that health psychologists would need to grapple with artificial intelligence as a central feature of their discipline would have seemed improbable. Today, it is inescapable. The algorithms are here—in our clinics, in our research designs, in the pockets of our patients—and they are reshaping the landscape of human health with a speed and scope that demands a response commensurate with the magnitude of the change.

The response I have advocated in this article is not one of resistance, nor of capitulation, but of philosophical depth. Health psychology has always been more than a branch of applied behavioral science. At its best, it has been a discipline that takes seriously the full humanity of the persons it serves—their embodiment, their subjectivity, their capacity for meaning-making, their vulnerability, their dignity. The algorithmic age does not diminish the importance of these commitments; it radicalizes them. It transforms them from comfortable professional platitudes into urgent philosophical necessities.

There are questions that no algorithm can answer. What does it mean to suffer? What does it mean to be present with someone who is in pain? What forms of life are worth promoting, and who has the right to decide? These are the questions that health psychology was created to address, and they remain as vital as ever—more vital, perhaps, precisely because the algorithmic transformation threatens to render them invisible. If health psychology loses sight of these questions in its eagerness to adopt the tools of artificial intelligence, it will gain efficiency and lose its soul. If it refuses the tools altogether, it will preserve its purity and lose its relevance.

The path I am advocating is harder than either of these alternatives. It requires a discipline willing to stand at the intersection of empirical science and philosophical reflection, to hold the tension between what can be measured and what can only be encountered, to insist on the irreducibility of human personhood while engaging honestly with technologies that challenge that irreducibility at every turn. This is the work of the next 15 years. It is the work that will determine whether health psychology remains a humanistic science—or becomes a branch of computational optimization wearing a human face.

The choice, at least for now, remains ours.

## 8 Future research directions

The framework advanced here suggests several concrete research priorities for the next decade of health psychology scholarship:

Phenomenological studies of algorithmic health identity: How do individuals experience and integrate health information generated by AI systems into their self-concept, illness narratives, and health behavior? Qualitative and mixed-methods research designs grounded in interpretive phenomenological analysis could illuminate the lived experience of the algorithmically informed patient.
Empirical disaggregation of therapeutic mechanisms: controlled studies that systematically isolate the contributions of relational presence, intervention structure, and algorithmic personalization to therapeutic outcomes would clarify what, precisely, is at stake in the transition to AI-mediated care.
Autonomy assessment in algorithmically mediated contexts: development and validation of measures capable of distinguishing between experienced autonomy and actual self-determination in digital health environments, drawing on both self-report and behavioral indicators.
Cross-cultural ontologies of AI and health: the disruptions described here may manifest very differently across cultural contexts with divergent conceptions of selfhood, illness, and care. Comparative research is essential to avoid the parochialism that has historically limited health psychology's global relevance.
Ethical frameworks for algorithmic health governance: interdisciplinary collaboration with philosophy, law, and computer science to develop governance structures that preserve moral accountability in AI-mediated health systems.
